# Efficiency of Stem Cell (SC) Differentiation into Insulin-Producing Cells for Treating Diabetes: a Systematic Review

**DOI:** 10.1155/2021/6652915

**Published:** 2021-02-25

**Authors:** Marzieh Nemati, GolamHossein Ranjbar Omrani, Bahareh Ebrahimi, Aliakbar Alizadeh

**Affiliations:** ^1^Endocrinology and Metabolism Research Center, Shiraz University of Medical Sciences, Shiraz, Iran; ^2^Shiraz Geriatric Research Center, Shiraz University of Medical Sciences, Shiraz, Iran; ^3^Department of Tissue Engineering and Applied Cell Sciences, School of Advance Medical Science and Technology, Shiraz University of Medical Sciences, Shiraz, Iran; ^4^Nanobiology and Nanomedicine Research Center, Shiraz University of Medical Sciences, Shiraz, Iran

## Abstract

Over the recent years, the use of stem cells has provided a new opportunity to treat various disorders including diabetes. Stem cells are unspecialized cells with a capacity for self-renewal and differentiation into more specialized cell types. Many factors contribute to the differentiation of SCs and thus play an important role in regulating the fate of stem cells. Accordingly, a wide range of protocols has been used to differentiate SCs to insulin-producing cells but the effectiveness of SC differentiation varies. The aim of this systematic review was to evaluate the results obtained from different studies on SC differentiation for higher efficacy to treat diabetes. This search was done in PubMed, Web of Science (WOS), and Scopus using keywords “insulin-producing cell (IPC),” “pancreatic B cell,” “insulin-secreting cell,” “stem cell,” “progenitor cells,” “mother cell,” and “colony-forming unit.” Among more than 3646 papers, 32 studies were considered eligible for more evaluations. The obtained results indicated that most of the studies were performed on the mesenchymal stem cells (MSCs) derived from different tissues as compared with other types of SCs. Different evaluations of *in vitro* studies as well as animal models supported their role in the recovery of diabetes. In the present review, we summarize and discuss recent advances in increasing the efficiency of SC differentiation using different materials, but despite the promising results of this systematic review, further studies are needed to assess the efficiency and safety of transplantation of these cells in diabetes recovery.

## 1. Introduction

Diabetes, as one of the most common chronic metabolic diseases, is considered a global health problem that threatens the well-being of a significant number of people across the world. Numerous data showed that the prevalence of diabetes is increasing [1], and the number of diabetics is expected to be doubled by 2025 [2]. Dietary control, regular blood sugar monitoring, oral antidiabetic drugs, and insulin injections are now the main diabetes treatments. These routines are not able to restore the precise and physiological function of beta cells and could cause long-term side effects [3, 4]. Islet transplantation is a new treatment that can significantly reduce the insulin dependence and restore glucose homeostasis; it is also considered as an effective treatment for diabetes. However, the lack of islets and suppression of the transplanted islets by the recipient's immune system have prevented the widespread use of this treatment [5], so researchers are motivated to find other treatments. The use of stem cells (SCs) and their differentiation into beta cells is a therapeutic strategy, which has received much attention in recent years. SCs are unspecialized cells with a capacity for self-renewal and differentiation into more specialized cell types [6]. Several factors may affect the differentiation of SCs such as extracellular matrix (ECM) proteins, cell-to-cell adhesion, cell-to-cell contact, and soluble factors [7]. A wide range of protocols have been used to differentiate SCs to insulin-producing cells. However, the efficiency of differentiation and the normal function of these newly formed *β*-cells, especially in response to glucose stimulation, are challengeable questions. The aim of this systematic review is to investigate the efficacy of differentiation of different SC sources and various interventions toward insulin-producing cells (IPCs). The results obtained from the present review could contribute to the assessment of translation of SC therapy in clinical trials.

## 2. Materials and Methods

### 2.1. Focused Question

This systematic review was performed to address the following question: “which intervention has the greatest effect on the differentiation efficacy of SCs into IPCs?”

### 2.2. Search and Study Selection

Keywords and abstract terms included ((Insulin-Producing Cell [Title/Abstract] OR Pancreatic *β*-cells [Title/Abstract]) OR Insulin Secreting Cell [Title/Abstract]) AND (((Stem Cell [Title/Abstract] OR Progenitor Cells [Title/Abstract]) OR Mother Cell [Title/Abstract]) OR Colony-Forming Unit [Title/Abstract]). The search strategy was applied to PubMed database, WOS, and Scopus, while focusing on the *in vitro* and animal model studies, and the inclusion criterion was the English language publications (by June 2020). The abstracts not published as full manuscripts, reviews, or the SC therapy studies for diseases other than diabetes were excluded. Data were collected from the full text articles as follows: (i) the source of SCs, (ii) type of the study (*in vitro* or *in vivo*), (iii) methods used for the evaluation of SC differentiation efficacy, and (iv) the obtained results.

## 3. Results

**Figure 1:**
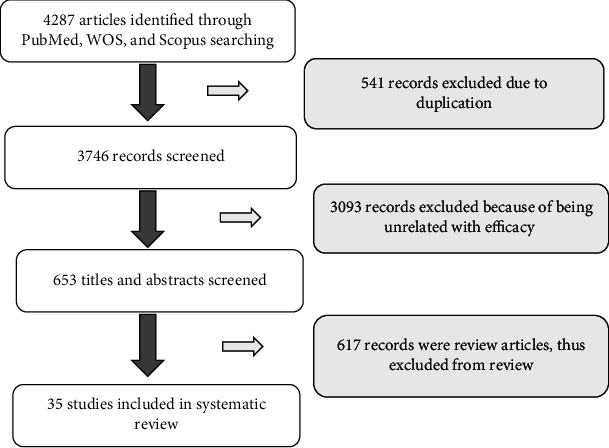
Literature search and study selection flowchart.

Search results and the characteristics of the included studies yielded 4287 studies. Among them, 3746 papers met all inclusion criteria and were selected after removing duplications. Moreover, 653 papers were reviewed and excluded (Figure 1).

By studying these article abstracts, only 35 articles included the results of the SC differentiation efficacy assay *in vitro* conditions (Table 1), and eight articles were related to the evaluation of treatment efficacy in animal models (Table 2). The extraction of data related to these studies is shown in Tables 1 and 2. Results revealed that the investigated SC cells have different sources. The SCs derived from embryo (ESCs) were used in twelve studies; bone marrow mesenchymal stem cells (BM-MSCs) were employed in six studies, whereas two studies investigated adipose tissue-derived mesenchymal stem cells (ADMSCs), four papers examined umbilical cord-derived mesenchymal stem cells (UCMSCs), and two articles discussed Warton jelly mesenchymal stem cells (WJMSCs) for the treatment of diabetes. Induced pluripotent stem cells (iPSCs) were used in five studies; only one study examined the pancreas stem cells and one study investigated human pluripotent stem cells (hPSCs). By all these source variations, only four different methods were used and these different methods can have an important effect on the SC differentiation efficacy. In the following section, these studies are discussed according to the method used.

### 3.1. Use of Transfection/Transduction for Differentiation of SCs into IPCs

During embryonic development, several transcription factors are involved in the beta cell differentiation. Pancreas/duodenum homeobox protein 1 (Pdx_1_) is one of the essential and crucial transcription factors for pancreatic development [8]. Paired box gene 4 (Pax_4_) is another crucial regulator of pancreas development, since the lack of Pax_4_ activity prevents the formation of mature pancreatic insulin-producing cells [9]. Moreover, neurogenin-3(Ngn_3_), MafA, and NeuroD are the main inducers of pancreatic endocrine-specific differentiation from its primordium [10, 11]. Therefore, different studies used various transcription factors to increase SC differentiation efficacy into IPCs. For example, Sun et al. (2006) transduced BMSCs using the eukaryotic expression vector containing Pdx-1. The immunocytochemical expression of genes in Pdx_1_(+)BMSCs were higher than that of Pdx_1_(−)BMSCs; also, glucose-induced insulin secretion from Pdx_1_(+)BMSCs was twofold more than that of Pdx_1_(−)BMSCs [9].

In two other studies, the researchers demonstrated that the overexpression of Pax_4_ using transfection could produce more mature IPCs from ESCs [9, 12]. Pax_4_ and Ngn_3_ transfection into murine ESCs also resulted in a significant upregulation of insulin genes (Ins1 and Ins2) and other pancreas-related genes and consequently generated efficient IPCs [13]. Also, expression of Pdx_1_ and MafA with either Ngn_3_ or NeuroD could significantly increase the differentiation efficiency of ESCs into IPCs. These observations were confirmed by qRT-PCR, immunocytochemistry, and ELISA assay [10]. Studies had shown that pdx_1_ and NKX_6.1_ gene transfection could increase the differentiation efficiency of induced pluripotent SCs to IPC and the obtained IPCs secreted significantly higher insulin and C-peptide levels [14]. Overexpression of Pax_4_ and MafA also resulted in ameliorating the differentiation efficiency of the pancreatic islet mesenchymal stem cells (PiMSCs) into IPCs [15]. The last study which used this method showed that the upregulation of Pax_4_ and Pdx_1_ could markedly enhance the differentiation of MSCs to form mature islet-like clusters and functional insulin-producing *β*-like cells [16].

### 3.2. Treatment Approaches Used for Differentiation of SCs into IPCs

Different combinations were used in eleven studies to increase the differentiation of SCs into IPCs. Hua et al. (2010) treated mouse ESCs by high concentration of exendin-4. The expression pattern of IPC markers showed that exendin-4 could improve the efficiency of differentiation of SCs toward the *β*-cell phenotype [8]. Pagliuca and Rezania found that using the modified differentiation protocol improved the differentiation efficiency of ESCs [17] and PSCs [18] into IPC. In addition, adding activin A to the cultural condition of hESCs could effectively produce beta cells [19]. Another study showed that BM-MSC treatment with pancreatic extract improved the differentiation efficiency and maturity of IPCs [20]. Zhang et al. (2013) demonstrated that using conophylline promoted induced pluripotent mesenchymal stem cell (iPMSC) differentiation into IPCs [21]. BMSCs [22], WJMSCs [23], and ADMSC [24] treatment with exendin-4 improved the differentiation outcome of these SCs to IPCs. Pezzolla et al. found that treating human embryonic stem cells (hESCs) with resveratrol improved the differentiation efficiency, produced numerous insulin-positive cells, and induced significantly higher Pdx_1_ expression [25]. Interestingly, addition of testosterone into the differentiation formula for pancreatic *β*-cells could increase the differentiation efficiency of human-induced pluripotent stem cells (hiPSCs) into IPCs from 12% to 35%. The administration of testosterone promoted the expression of key genes associated with *β*-cell differentiation including Ngn_3_, NeuroD_1_, and INS [26].

### 3.3. Coculture Method Used for Differentiation of SCs into IPCs

Various studies have used different substances in SC coculturing in order to increase the differentiation efficacy of SCs into IPCs. For instance, using endothelial cell coculture for enhancing ESC differentiation has been reported only in one study, revealing that endothelial cells enhanced embryonic stem cell differentiation to pancreatic progenitors and IPCs through bone morphogenetic protein (BMP) signaling [27]. Bea et al. (2015) cultured pluripotent SCs in the presence of mature islet cells. They found that the paracrine effects of these cells could lead to an increase in the differentiation efficiency of the pancreatic endoderm (PE) cells into mature IPCs [28]. A study performed by Yilmaz et al. (2015) revealed that the ESC coculture with mouse pancreatic islets resulted in higher differentiation efficacy toward IPCs [29].

### 3.4. Use of Extracellular Matrix Proteins for Differentiation of SCs into IPCs

In 2007, Farrokhi et al. evaluated the effect of extracellular matrices (ECMs) on their differentiation to IPCs. RT-PCR and also secretory function analysis of their finding showed higher expression of *β*-cell-specific markers including insulin I, insulin II, Slc_2_a_2_, and more insulin secretion in the matrigel-coated plate group [30]. In two other studies, using fibronectin, laminin [31], and laminin 411 [32] promoted the differentiation of MSCs into insulin-producing cells and had upregulated insulin expression at both mRNA and protein levels. Rasmussen et al. (2015) also reported that treating SCs by ECM proteins in a particular collagen type I with fibronectin could improve the differentiation of human ESCs toward definitive endoderm [33].

### 3.5. Use of *In Vivo* Microenvironmental Simulation for Differentiation of SCs into IPCs

Seyedi et al. (2015) used a 3D culture condition (hanging drop) to differentiate hUCMs into IPC. RT-PCR and immunohistochemistry analysis showed higher expression of insulin protein and insulin production in hanging drops than the monolayer group [34]. RT-PCR, immunofluorescence, and radioimmunoassay (RIA) in another study revealed the improvement in the differentiation efficiency of BMSCs toward IPCs under a nonadherent state [35]. Evaluation using a 3D culture to differentiate ADMSCs [36, 37], BMSCs [36, 37], WJMSCs [33, 38], and human umbilical mesenchymal stem cells (hUCMSCs) [39] by different evaluation methods, such as RT-PCR, immunofluorescence, RIA, and ELISA assay, showed more than a threefold increase in the differentiation efficacy compared to the 2D experimental culture cell usage.

### 3.6. Animal Studies Performed for the Treatment of Diabetes by Transplantation of IPC Differentiated from SCs

The efficacy of SC-derived IPC therapy on diabetes was evaluated in eight different animal studies. Xie et al. transplanted IPCs derived from MSCs treated with pancreatic extract under the right kidney capsule of streptozotocin- (STZ-) induced diabetic rats to evaluate the ability of these cells in the treatment of diabetes. They observed that IPCs were able to release more insulin in a glucose-dependent manner and ameliorate the diabetic conditions of STZ-treated rats better [20]. In other studies, the hESC-derived islet-like clusters were used and injected under the kidney capsule or the epididymal fat pad could transiently normalize glycaemia in diabetic mice ([25], Kroon et al. 2008, [17]). Other studies investigated the effect of differentiation efficacy of pancreatic islet-like cell clusters derived from hBMSCs under nonadherent induction by transplanting differentiated cells to diabetic mice. They found that these cells normalized hyperglycemia in diabetic mice [35]. Another study on diabetic nude mice indicated that transplantation diabetic mice with IPCs derived from pancreatic stem cells cocultured with islet cells exhibited restored euglycemia and improved glucose tolerance [28]. Qu et al. (2014) also revealed that administration of laminin 411-induced IPCs rapidly and significantly downregulated fasting blood glucose levels, significantly reduced the HbA_1_c concentration, and markedly improved the symptoms and survival of diabetic rats [32]. Pagliuca and his colleagues found that transplantation of IPCs derived from PSCs by using sequential modulation of multiple signaling pathways ameliorated hyperglycemia in diabetic mice [18].

## 4. Discussion

This study is the first systematic review about the efficacy of SC differentiation toward IPCs in restoration of diabetes. This review included the results of *in vitro* differentiation and animal studies. The various sources of SCs used in these studies and their different evaluation methods highlight the advantage of comparing the obtained results with each other. In most studies, BM-MSCs and ESCs were used under variable conditions to differentiate into IPCs for assessing the best differentiation efficacy in diabetes therapy. The results of these studies indicated that the transfected stem cells with IPC key transcription factors, including PDX_1_, PAX_4_, MafA, NKX_6.1_, and NeuroD, could improve the differentiation efficacy of SCs into IPCs. As the results of various studies have shown, the use of these essential factors is considered as an effective factor in SC differentiation into IPCs ([8, 9, 12, 13], Xie, Wang et al. 2013, [14, 15]). Furthermore, it appears that the treatment of SCs with different factors plays a key role in the improvement of differentiation efficacy and subsequently on the therapeutic outcome.

**Figure 2:**
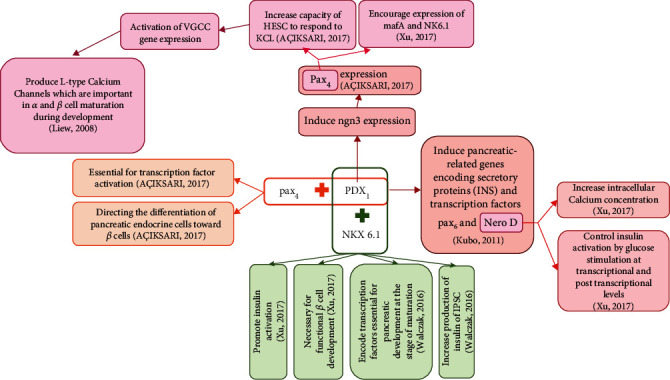
Summary of different pathways increasing beta cell differentiation.

The comparison of the obtained results indicated that the type of treatments could influence the efficacy of differentiation through diverse pathways (Figure 2). Some factors may improve it via stimulating the expression of islet-associated genes (Pdx_1_ and insulin), leading to increased insulin release upon glucose challenge, as demonstrated by Hua et al. (2010) [8], Nejad-Dehbashi et al. (2013) [22], and others [14, 36, 37]. Rat pancreatic extract has been used as a differentiation inducer because it contains pancreatic development and differentiation proteins that could improve the differentiation efficacy of BM-MSCs and also ameliorate the diabetic conditions of streptozotocin- (STZ-) treated rats [20]. In addition, the result of another study showed that conophylline stimulated iPMSC proliferation and promoted their potential differentiation into an islet-like cluster [21]. In three studies, the coculture technique was used to increase the differentiation efficacy of SCs into IPCs [27–29]. The results obtained from these three studies indicated that the differentiation of SCs into insulin-producing cells under coculture conditions with endothelial cells or islet cells were effectively increased. Additionally, an *in vivo* study showed that the diabetic mice transplanted with differentiated SC coculturing with islet cells exhibited restored euglycemia and improved glucose tolerance [28].

Several research groups tried to examine the effect of different extracellular matrix proteins on stem cell differentiation efficacy. The results obtained by Hsiao-Yun et al. indicated that IPC differentiation by MSCs could be enhanced by adding ECM and these stimulatory effects were mediated through activation of Akt and ERK pathways [31]. The use of laminin 411 by Huiting et al. (2014), as a differentiation inducer of IPCs from UC-MSCs via the Pdx_1_ and Ngn_3_ signaling pathways, efficiently improved the differentiation, symptoms, and survival of diabetic rats [32]. Another study performed by Rasmussen et al. (2015) demonstrated that collagen I could effectively differentiate the embryonic SCs into functional *β*-cells [33]. The evaluation of stem cell differentiation efficacy on 3D culture environments has been performed in five studies [34–37, 39]. A 3D culture is advantageous to imitate the *in vivo* microenvironment by enhancing cell-cell and cell-matrix interactions and the subsequent cell signaling [40, 41]. In line with this fact, the results obtained by Zhang (2014), Khorsandi et al. (2014), Khorsandi et al. and (2015) showed that nonadherent induction [35], collagen/hyaluronic acid (Col/HA) scaffold [36, 37], and FG scaffolds [36, 37] could greatly promote SCs to form pancreatic islet-like cell clusters and improve the differentiation efficiency of SCs toward IPCs.

## 5. Conclusion

In summary, the results obtained from this systematic review provide better understanding about the efficacy of SC differentiation into IPCs for treating diabetes. The obtained results showed that the IPCs derived from SCs by imitating the *in vivo* microenvironment and using 3D culture showed a better efficacy than the other methods of differentiation to restore diabetes. However, the obtained results indicate that more evaluation is needed about the efficacy of SC differentiation into IPCs for the treatment of diabetes.

## Figures and Tables

**Table 1:** Overview of in vitro differentiation efficacy of SCs into IPC.

Cell source	Intervention (s)	Outcome	Author (year)
BM-MSCs	Pdx-1 transfection	The results demonstrated that Pdx-1 could improve differentiation efficacy of BMSCs to IPC and increase gene expression.	Sun et al. [9]
BM-MSCs	Fibronectin and laminin	The data showed pancreatic differentiation enhancement.	Lin et al. [31]
BM-MSCs	Pancreatic tissue extract	Pancreatic tissue extract effectively promoted differentiation efficiency and maturity of IPCs.	Xie et al. [20]
BM-MSCs	Exendine-4	Results demonstrated EX-4 significant increase in differentiation of IPCs from RAT-BM-MSCs.	Dehbashi-Nejad et al. (2014)
BM-MSCs	Nonadherent state	Nonadherent induction greatly improved the differentiation efficiency of BMSCs towards IPCs.	Zhang et al. [35]
BM-MSCs	Fibrin glue (FG), Pdx-1, Glut-2, (3D culture)	FG scaffold enhanced the differentiation of IPCs from rat BM-MSCs.	Khorsandi et al. [24]
WJ-MSCs	Exendin-4	Incorporating exendin-4 significantly improved the differentiation outcome of WJ-MSCs into IPCs.	Kassem et al. [23]
WJ-MSCs	Decellularized liver scaffolds (3D culture)	3D scaffold improved differentiation of WJMSCs to IPCs.	Zhou et al. [38]
UC-MSCs	Laminin 411	Laminin 411 significantly enhanced differentiation efficiency of IPCs from MSCs.	Qu et al. [32]
UC-MSCs	Hanging drops (3D culture)	Differentiation of hUCMs into insulin-secreting cells was more efficient in 3D culture than 2D culture.	Seyedi et al. [34]
UC-MSCs	Fibrin scaffold	Cells in fibrin scaffold 3D culture system were much more efficient than those in 2D conventional culture system.	Seyedi et al. [39]
UC-MSCs	PAX4 and PDX1	PAX4 promotes the PDX1-induced differentiation of MSCs into functional *β*-cells.	Xu et al. [16]
AD-MSCs	Collagen/hyaluronic acid (Col/HA) scaffold (3D culture), PDX1	The results showed that COL/HA scaffold enhanced the differentiation of IPCs from rat AMSCs.	Khorsandi et al. [36, 37]
AD-MSCs	Exendine-4	EX-4 significantly enhanced the differentiation of ADMSCs into IPCs.	Khorsandi et al. [24]
iPMSCs	Conophylline	Data showed that conophylline promoted the iPMSC differentiation into islet-like clusters.	Zhang et al. [35]
iPSC	PDX-1 and NKX6.1 transfection	Efficiency of differentiation of iPSC to IPC increased by concurrent expression of *PDX1* and *NKX6.1.*	Walczak et al. [14]
iPSCs	Testosterone	The addition of testosterone into routine differentiation formula for pancreatic beta cells increased differentiation efficiency from 12% to 35%.	Liu et al. [26]
PSCs	Sequential modulation of multiple signaling pathways	The stem cell-derived beta cells expressed markers found in mature beta cells and secreted quantities of insulin comparable to adult beta cells in response to multiple sequential glucose challenges in vitro.	Pagliuca et al. [18]
PSCs	Coculture with mature islets	Results suggested that mature islet cells could increase the differentiation efficiency of PE cells into mature IPCs via paracrine effects.	Oh et al. [28]
ESCs	Activin A	Results indicated that the hES cell-derived insulin-expressing cells contained many cell components critical to *β*-cell function.	D'Amour et al. [19]
ESCs	Pax4 nucleofection	Data demonstrated that pax4 increased differentiation of ESCs.	Lin et al. (2007)
ESCs	Pax4 overexpression	Results showed that overexpression of Pax4 enhanced generation of cells resembling pancreatic *β*-cells.	Liew et al. [12]
ESCs	Extracellular matrix	Results showed that matrigel-coated plates increased the differentiation efficacy of ESCs into IPCs (higher expression insulin I, insulin II, Slc_2_a_2_, and more insulin secretion).	Farrokhi et al. [30]
ESCs	Exendin-4 transfection	Exendin-4 improved the efficiency of ESC differentiation toward the beta cell phenotype.	Li et al. [8]
ESCs	Ngn3 and Pdx1 overexpression	This combination dramatically increased the differentiation efficacy.	Kubo et al. [13]
ESCs	Coculture with endothelial cells	Results demonstrated that differentiation of ESCs to insulin-producing cells enhanced by coculture with ECs.	Talavera et al. [27]
ESCs	Pdx1, MafA with either Ngn3 or NeuroD overexpression	Treatment with ESCs promoted differentiation of ES cells into insulin-secreting cells.	Xu et al. [10]
hESCs	Using a combination of reagents, including an ALK5 inhibitor, BMP receptor inhibitor, and thyroid hormone (T3)	The results showed that highly differentiated cells displayed certain key characteristics of mature beta cells.	Rezania et al. [17]
ESCs	Collagen type 1	Collagen I induced more rapid and consistent differentiation of stem cells to definitive endoderm.	Rasmussen et al. [33]
ESCs	Resveratrol	RSV treatment enhanced efficient differentiation of hESCs into *β*-cell-like cells.	Pezzolla et al. [25]
ESCs	Coculture with mouse pancreatic islets	Results demonstrated higher differentiation efficiency in the coculture group.	Yilmaz et al. [29]
Pancreatic islet-derived mesenchymal stem cells	MafA, Pax4, and Ngn3 transfection	Cotransfection of these factors improved differentiation efficiency of the stem cells into insulin-secreting cells.	Aciksari et al. [15]

Bone marrow mesenchymal stem cells (BM-MSCs); umbilical cord mesenchymal stem cell (UC-MSC); mesenchymal stem cells isolated from Wharton's jelly of umbilical cord (WJ-MSCs); adipose-derived mesenchymal stem cell (AD-MSC); embryonic stem cells (ESCs); induced pluripotent stem cells (iPSCs); immortalized pancreatic mesenchymal stem cells (iPMSCs); pluripotent stem cells (PSCs).

**Table 2:** Overview of in vivo differentiation efficacy of SCs into IPC.

Cell source	Intervention (s)	Type of animals	Transplanted site	Methods of transplantation	Observation period	Outcome	Author (year)
BM-SCs	Transplant of IPCs derived from BM-SCs in nonadherent condition	Diabetic mice	Testis	Surgery and injection	3 months	Data showed that IPCS transplantation could normalize the blood glucose levels.	Zhang et al. [35]
BM-SCs	Transplant of IPCs derived from MSCs treating with pancreatic extract	Diabetic rat	Under kidney capsule	Surgery and injection	35 days	Transplantation of these cells better ameliorated the diabetic conditions.	Xie et al. [20]
UC-MSCs	Intravenous injection of IPCs produced in the presence of laminin 411 in culture condition	Diabetic rat	Tail vein	Injection	4 weeks	The results showed that administration of these cells markedly improved the symptoms of diabetes.	Qu et al. [32]
PSCs	Transplant of IPCs derived from PSCs coculturing with islet cells	Diabetic mice	Under kidney capsule	Surgery and injection	90 days	Transplantation of differentiated cells restored euglycemia and improved glucose tolerance.	Oh et al. [28]
PSCs	Transplant of IPCs derived from PSCs by using sequential modulation of multiple signaling pathways	Mice	Under kidney capsule	Surgery and injection	112 days	Transplantation of these cells ameliorated hyperglycemia in diabetic mice.	Pagliuca et al. [18]
iPSCs	Transplant of IPCs derived from hiPSC treating with resveratrol	Diabetic mice	Under kidney capsule	Surgery and injection	6 weeks	Transplantation with differentiated cells could normalize glycemia.	Daniela et al. (2015)
hESCs	Transplant of IPCs derived from hESCS in modified differentiation protocol	Diabetic mice	Epididymal fat pads (EFP)	Surgery	200 days	The hES cell-derived endocrine cells are functionally very similar to adult human islets.	Kroon et al. (2008)
hESCs	Transplant of IPCs derived from hESCS in modified differentiation protocol	Diabetic mice	Under kidney capsule	Surgery and injection	120 days	The results showed that transplantation of these cells rapidly reversed diabetes.	Rezania et al. [17]
